# Supplementary material for: Dissecting apicoplast functions through continuous cultivation of Toxoplasma gondii devoid of the organelle
Source: Nat Commun. 2025 Mar 1;16:2095. doi: 10.1038/s41467-025-57302-x (PMC11873192; doi:10.1038/s41467-025-57302-x)

Supplementary Information File

Dissecting apicoplast functions through continuous cultivation of *Toxoplasma gondii* devoid of the organelle

Min Chen, Szilamér Gyula Koszti, Alessandro Bonavoglia, Bohumil Maco, Olivier von Rohr, Hong-Juan Peng, Dominique Soldati-Favre and Joachim Kloehn

* Corresponding authors: Hong-Juan Peng, Dominique Soldati-Favre, Joachim Kloehn

Floriapeng@hotmail.com, Dominique.soldati-favre@unige.ch, Joachim.Kloehn@unige.ch

This PDF file includes:

- Supplementary Figures 1-7 and their legends
- List of abbreviations related to main Figure 7c
- Uncropped blots and images related to Supplementary Figures

Supplementary Fig. 1: Manipulation of the FabG, ATS1 and LipA loci, and localization of these proteins.

a Cartoon schematic depicting the strategy for constructing conditional knockdown strains for genes of interest (GOI) mediated by Cre-*LoxP* and a U1 destabilization domain in DiCre expressing parasites. The indicated primers are listed in Supplementary data 1. **b** Integration PCRs from genomic DNA extracts of DiCre parental line, iKD FabG-Ty, iKD ATS1-Ty and iKD LipA-Ty. **c** Indirect immunofluorescence assays (IFAs) of iKD FabG-Ty parasites, stained with anti-Ty and anti-Cpn60 (apicoplast lumen marker) antibodies. Source data are shown at the end of this file.

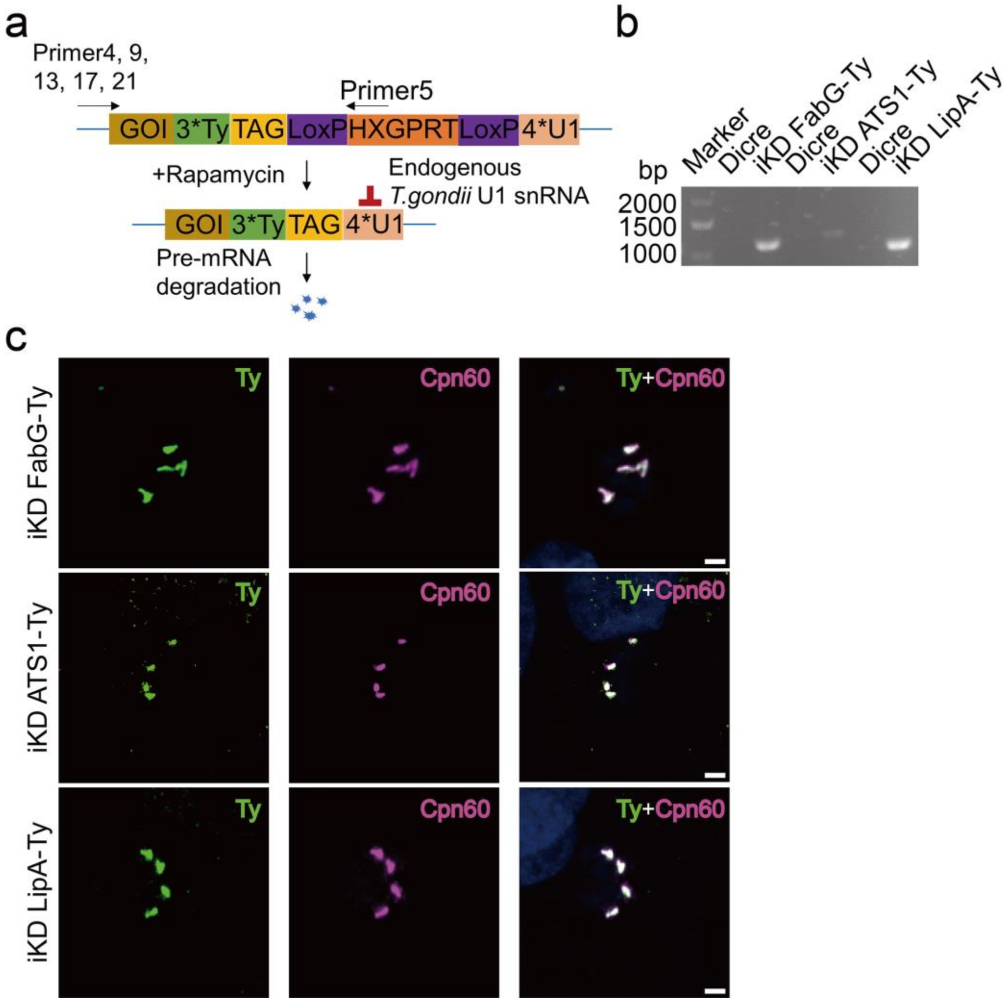

Supplementary Fig. 2: High serum concentrations rescue defects in the apicoplast-localized fatty acid and lipid synthesis pathways.

a Plaque assays of DiCre, iKD FabG-Ty, iKD ATS1-Ty and iKD LipA-Ty parasites in medium containing rapamycin (Rapa) or not and different concentration of fetal bovine serum (FBS) (**a**). **b-e** Quantification of plaque sizes (n = 3) for DiCre (**b**), iKD FabG-Ty (**c**), iKD ATS1-Ty (**d**) and iKD LipA-Ty (**e**) parasites. **f** Representative indirect immunofluorescence assays (IFA) showing partial loss of the apicoplast in iKD FabG-Ty parasites after 72 h of Rapa treatment, based on staining with anti-GAP45 (pellicle marker), and anti-ATrx1 (apicoplast membrane marker) antibodies. **g** Representative IFA showing partial loss of the apicoplast in iKD FabG-Ty parasites after 72 h of Rapa treatment, based on anti-actin (cytosol marker), and anti-Cpn60 (apicoplast lumen marker) as well as DAPI staining (DNA – nuclei and apicoplasts). **h** Quantification of apicoplast loss in DiCre and iKD FabG -Ty parasites in presence or absence of Rapa (72 h), based on anti-ATrx1, anti-Cpn60 and DAPI staining. **i** Apicoplast loss in DiCre, iKD FabG-Ty, iKD ATS1-Ty and iKD LipA-Ty parasites in presence or absence of Rapa and in normal medium or medium supplemented with 100 µM C14: 0 or C16:0 (72 h Rapa and FA supplementation) (n = 3). All bar graphs show the means of three independent experiments, with error bars indicating the standard deviation. In b-e, dots represent means of three experiments, averaging a minimum of 10 plaques per experiment. Student's two-sided t-tests compare the indicated conditions. p-values are given and were considered significant at $p < 0.05$. Scale bars in f and g: 2 µm. Source data are provided as a Source Data file.

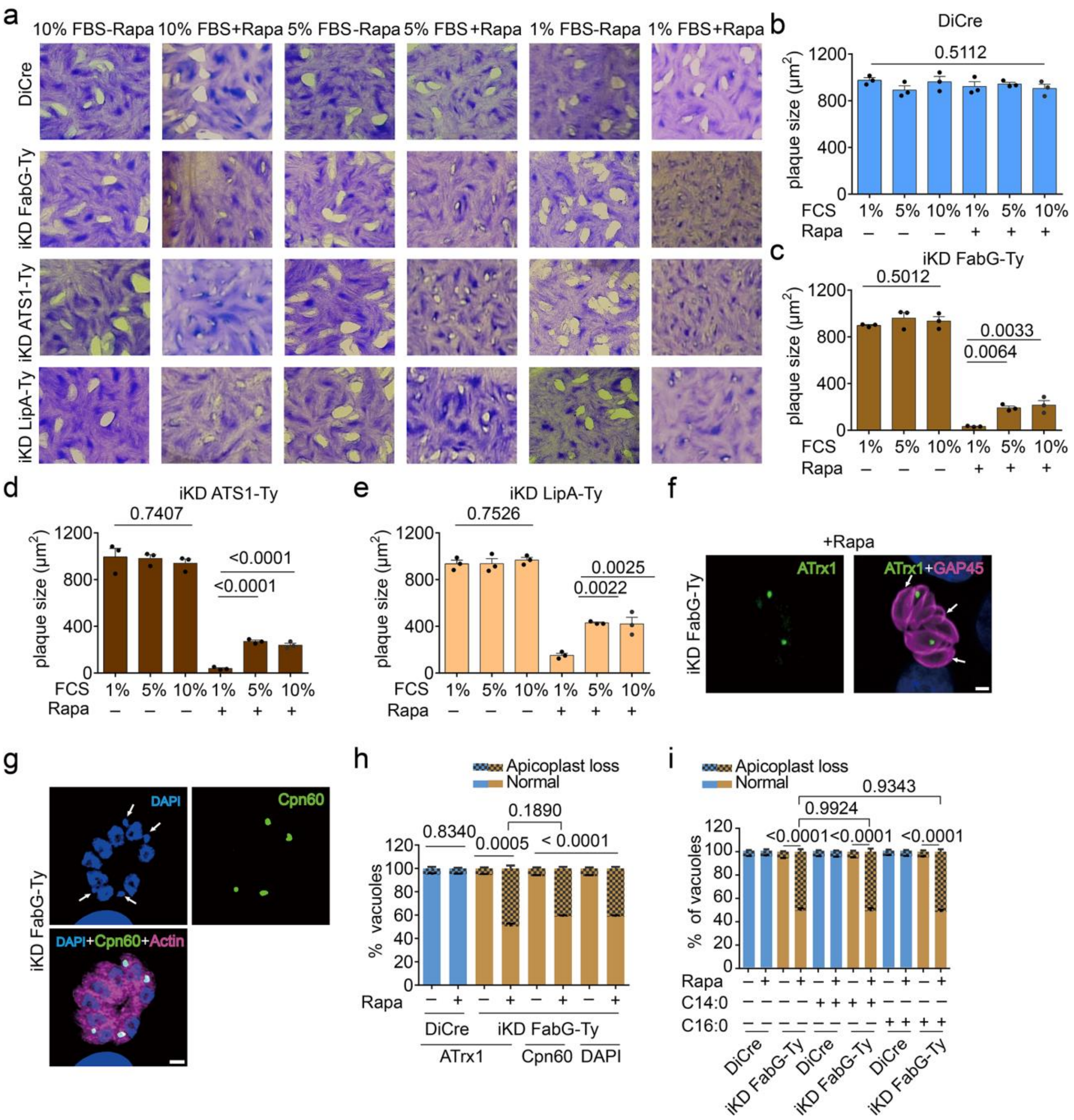
a
10% FBS-Rapa
10% FBS+Rapa
5% FBS-Rapa
5% FBS+Rapa
1% FBS-Rapa
1% FBS+Rapa
DiCre
iKD FabG-Ty
iKD ATS1-Ty
iKD LipA-Ty
b
DiCre
0.5112
plaque size (μm²)
FCS 1% 5% 10% 1% 5% 10%
Rapa – – – + + +
c
iKD FabG-Ty
0.5012
0.0033
0.0064
plaque size (μm²)
FCS 1% 5% 10% 1% 5% 10%
Rapa – – – + + +
d
iKD ATS1-Ty
0.7407
<0.0001
<0.0001
plaque size (μm²)
FCS 1% 5% 10% 1% 5% 10%
Rapa – – – + + +
e
iKD LipA-Ty
0.7526
0.0025
0.0022
plaque size (μm²)
FCS 1% 5% 10% 1% 5% 10%
Rapa – – – + + +
f
+Rapa
ATrx1
ATrx1+GAP45
iKD FabG-Ty
g
DAPI
Cpn60
DAPI+Cpn60+Actin
iKD FabG-Ty
h
Apicoplast loss
Normal
0.8340
0.1890
0.0005
< 0.0001
% vacuoles
Rapa – + – + – + – +
DiCre
iKD FabG-Ty
ATrx1
Cpn60
DAPI
i
Apicoplast loss
Normal
0.9343
0.9924
<0.0001
<0.0001
<0.0001
% of vacuoles
Rapa
C14:0
C16:0
DiCre
iKD FabG-Ty
DiCre
iKD FabG-Ty
DiCre
iKD FabG-Ty

Supplementary Fig. 3: Characterization of apicoplast-localized heme and isoprenoid synthesis pathways.

a Integration PCRs from genomic DNA extracts of DiCre parental line, iKD PBGD-Ty and iKD IspH-Ty parasites. **b** Indirect immunofluorescence assays (IFAs) of iKD PBGD-Ty and iKD IspH parasites, stained with anti-Ty and anti-Cpn60 (apicoplast lumen marker) antibodies. **c, d** Intracellular growth assay of DiCre and iKD PBGD-Ty (**c**) or iKD IspH-Ty (**d**) parasites in presence or absence of rapamycin (Rapa). Data reflects intracellular growth over 24 h and a total duration of 72 h Rapa treatment (n = 3). **e, f** Quantification of apicoplast loss in DiCre and iKD PBGD-Ty (**e**) or iKD IspH-Ty (**f**) parasites in presence or absence of Rapa (72 h), as determined by ATrx1 staining. **g** Genomic PCR validating integration of the MVA-HA cassette into the UPRT locus of iKD IspH-Ty parasites. **h, i** Plaque assays of iKD IspH-MVA-HA parasites cultured in medium +Rapa, supplemented with different concentration of mevalonolactone (MVL) (**h**) and quantification of plaque sizes (**i**) (n = 3). Images in a, b, g and h are representative of three independent experiments. All bar graphs show the means of three independent experiments, with error bars indicating the standard deviation. In i, dots represent the means of three experiments, averaging at least 10 plaques per experiment. Student's two-sided t-tests compare the indicated conditions. p-values are given and were considered significant at $p < 0.05$. Scale bars in b: 2 µm. Source data are provided as a Source Data file and at the end of this file.

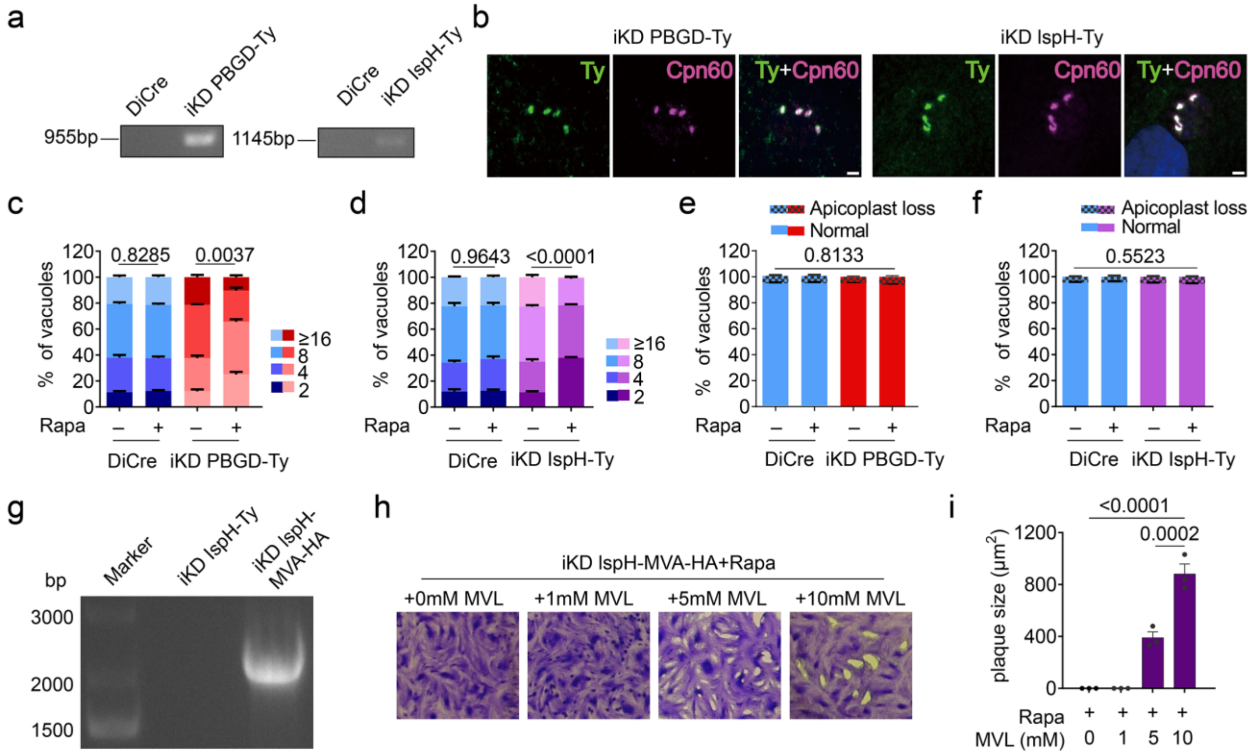
a
DiCre
iKD PBGD-Ty
955bp
DiCre
iKD IspH-Ty
1145bp
b
iKD PBGD-Ty
Ty
Cpn60
Ty+Cpn60
iKD IspH-Ty
Ty
Cpn60
Ty+Cpn60
c
% of vacuoles
0.8285
0.0037
≥16
8
4
2
Rapa
DiCre
iKD PBGD-Ty
d
% of vacuoles
0.9643
<0.0001
≥16
8
4
2
Rapa
DiCre
iKD IspH-Ty
e
Apicoplast loss
Normal
% of vacuoles
0.8133
Rapa
DiCre
iKD PBGD-Ty
f
Apicoplast loss
Normal
% of vacuoles
0.5523
Rapa
DiCre
iKD IspH-Ty
g
bp
Marker
iKD IspH-Ty
iKD IspH-MVA-HA
3000
2000
1500
h
iKD IspH-MVA-HA+Rapa
+0mM MVL
+1mM MVL
+5mM MVL
+10mM MVL
i
plaque size (μm²)
<0.0001
0.0002
Rapa
MVL (mM)
0
1
5
10

Supplementary Fig. 4: Generation and characterization of apicoplast-less *T. gondii* (RH-MVA-HA^{-Apico}).

a Integration PCR from genomic DNA extracts of RH parental line, and RH-MVA-HA parasites. **b** Indirect immunofluorescence assays (IFAs) of RH and RH-MVA-HA parasites, stained with anti-HA and anti-GAP45 antibodies. **c** Western blot of RH and RH-MVA-HA parasite protein lysates. Membranes were probed with anti-HA antibodies and anti-catalase as loading control. **d**, **e** Plaque assays of RH-MVA-HA parasites cultured in medium with actinonin (40 µM) and supplemented with apicoplast rescue medium (ARM) or ARM lacking individual or paired metabolites as indicated (– indicates the removed metabolites, note that the ARM control condition is re-plotted from the main Figure panel 5i) (**d**) and the corresponding plaque size quantification (**e**). **f** Plaques of lysis generated by 250 RH-MVA-HA or RH-MVA-HA^{-Apico} parasites from the mouse inoculum. **g** Western blot of HFF (control) and *T. gondii* parasite (RH) extracts probed with mouse serum from mice infected with RH-MVA-HA or RH-MVA-HA^{-Apico} parasites. Images in a-d, f and g are representative of three independent experiments. Bar graphs in e show the means of three independent experiments, with error bars indicating the standard deviation. Dots represent the means of each experiment, averaging 10 or more plaques per experiment. Student's two-sided t-tests compare the indicated conditions. p-values are given and were considered significant at $p < 0.05$. Scale bars in b: 2 µm. Source data are provided as a Source Data file and at the end of this file.

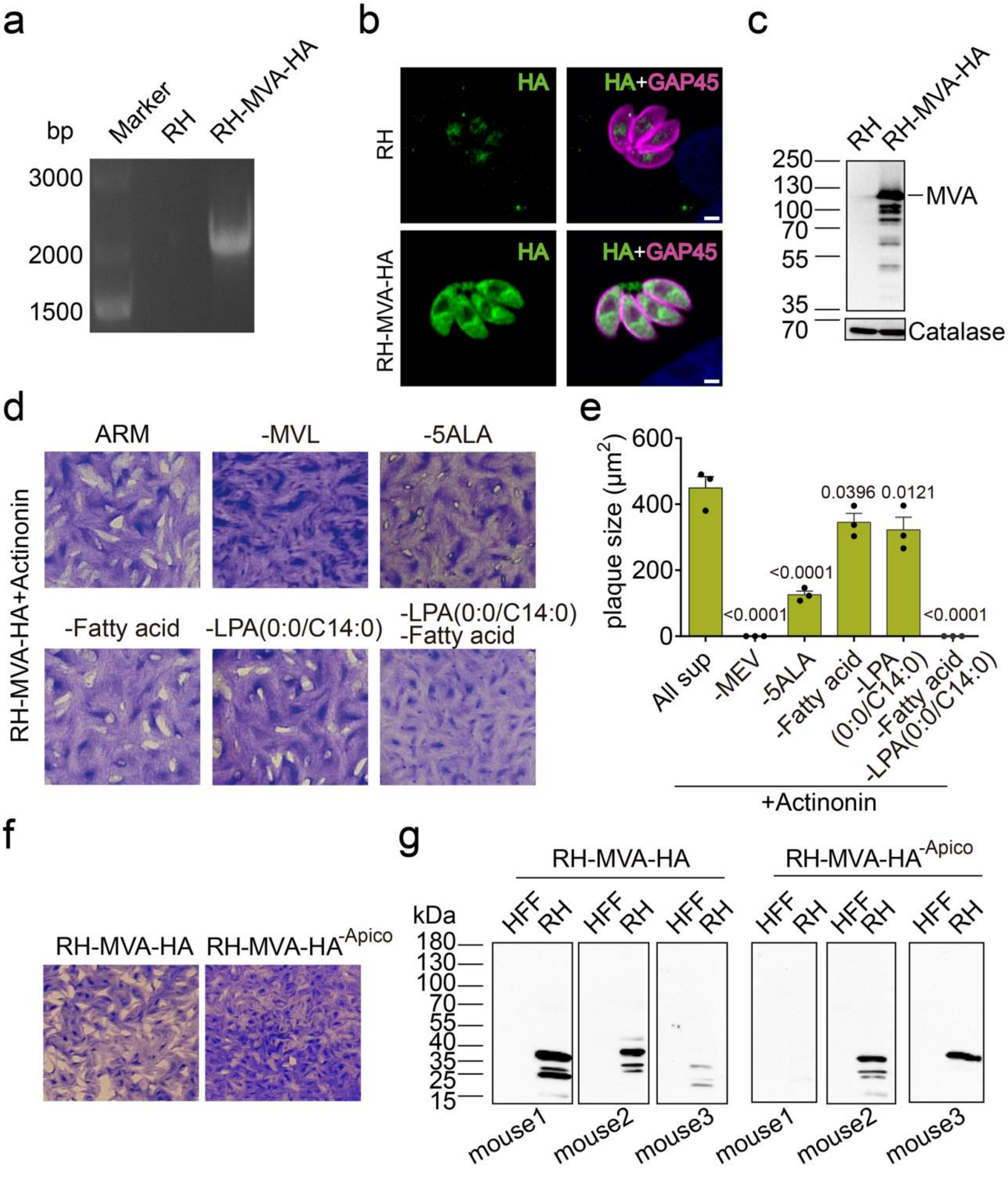

a
bp
Marker
RH
RH-MVA-HA
3000
2000
1500
b
HA
HA+GAP45
RH
RH-MVA-HA
c
RH
RH-MVA-HA
250
130
100
70
55
35
70
MVA
Catalase
d
ARM
-MVL
-5ALA
-Fatty acid
-LPA(0:0/C14:0)
-LPA(0:0/C14:0)
-Fatty acid
RH-MVA-HA+Actinonin
e
plaque size (μm²)
600
400
200
0
0.0396
0.0121
<0.0001
<0.0001
<0.0001
All sup
-MEV
-5ALA
-Fatty acid
-LPA (0:0/C14:0)
-Fatty acid -LPA(0:0/C14:0)
+Actinonin
f
RH-MVA-HA
RH-MVA-HA^-Apico
g
RH-MVA-HA
RH-MVA-HA^-Apico
kDa
HFF
RH
180
130
100
70
55
40
35
25
15
mouse1
mouse2
mouse3

Supplementary Fig. 5: Electron micrograph images of apicoplast-less *T. gondii*.

a Electron micrograph images of a single section through vacuoles containing eight parasites of the control line (RH-MVA-HA) and parasites presumed devoid of an apicoplast (RH-MVA-HA^{-Apico}). Apicoplasts detected in a are indicated by white arrows (only seen in RH-MVA-HA, left panel). **b** Consecutive sections (20) through the apical end a single RH-MVA-HA^{-Apico} parasite, revealing absence of any apicoplast.

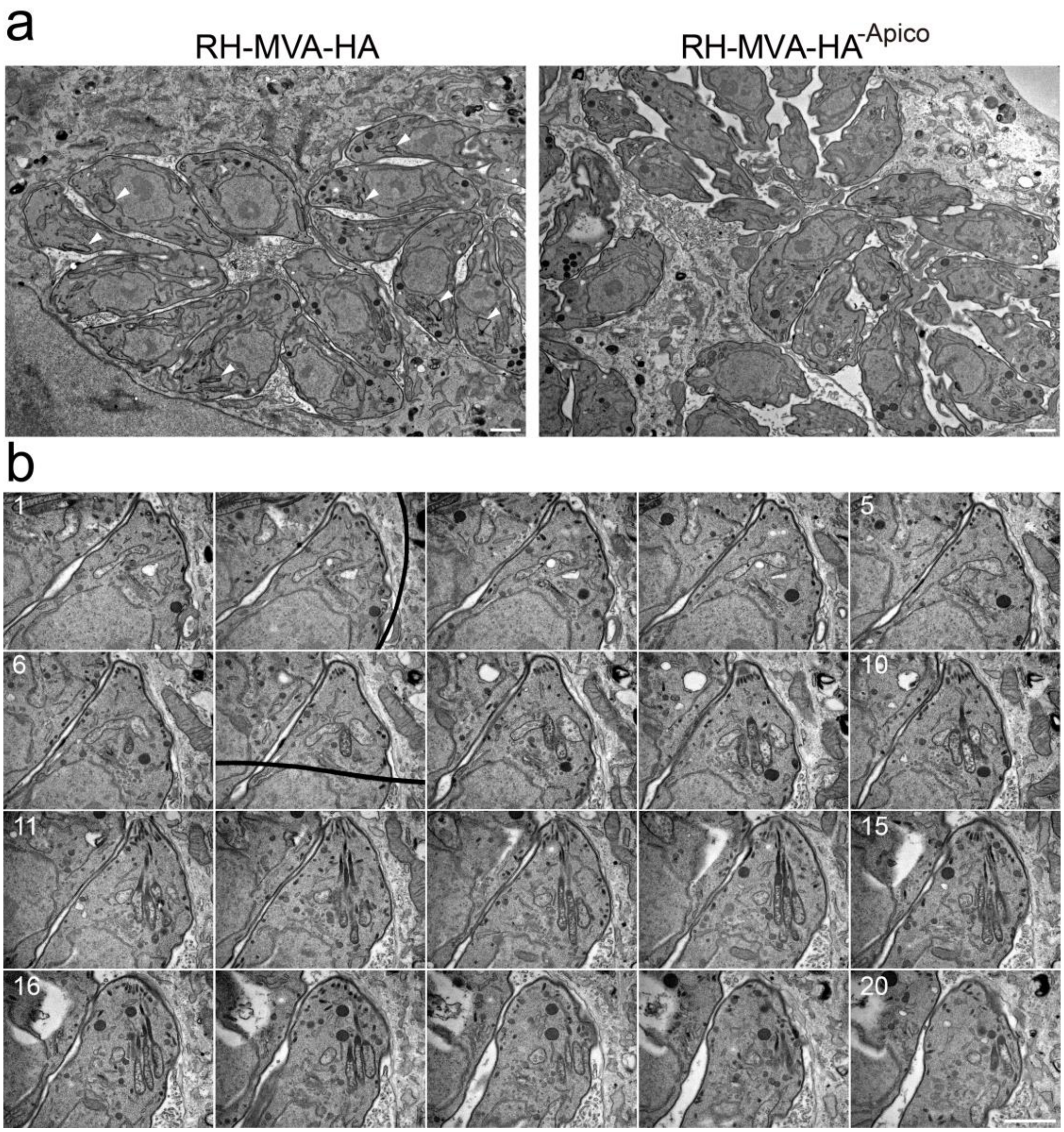

Supplementary Fig. 6: Deletion of *UROS* in *T. gondii* lacking an apicoplast.

a Two-guide CRISPR/Cas9 RNA strategy for deleting the locus of the uroporphyrinogen III synthase (UROS) and replacing it with a dihydrofolate reductase (DHFR) resistance cassette. **b** Genomic PCR confirming the deletion of the *UROS* locus and the insertion of the DHFR resistance cassette in RH-MVA-HA^{-Apico} parasites (here: −Apico). Amplicon sizes are given and see schematic indicating amplicons in a. Source data are shown at the end of this file.

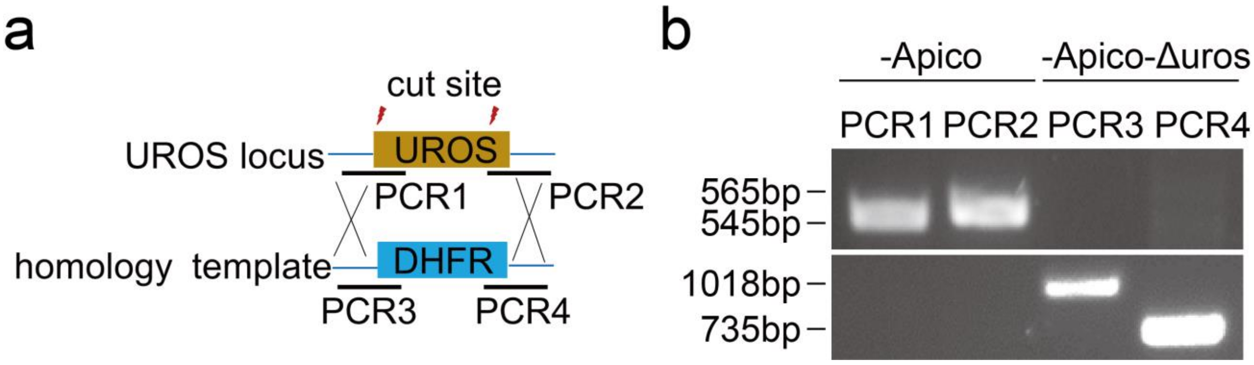

Supplementary Fig. 7: Additional phenotyping of FtsH1.

a Intracellular growth assay of DiCre and iKD FtsH1-Ty parasites. Data reflects intracellular growth over 24 hours and total duration of rapamycin (Rapa) treatment of 72 hours (n = 3). **b** Quantification of apicoplast loss based on ATrx1 staining in DiCre and iKD FtsH1-Ty parasites, following treatment with Rapa for varying durations (n = 3). All bar graphs show the means (bars) of three independent experiments. Error bars indicate the standard deviation. Student's two-sided t-tests compare the indicated conditions. p-values are given and were considered significant at $p < 0.05$. Source data are provided as a Source Data file.

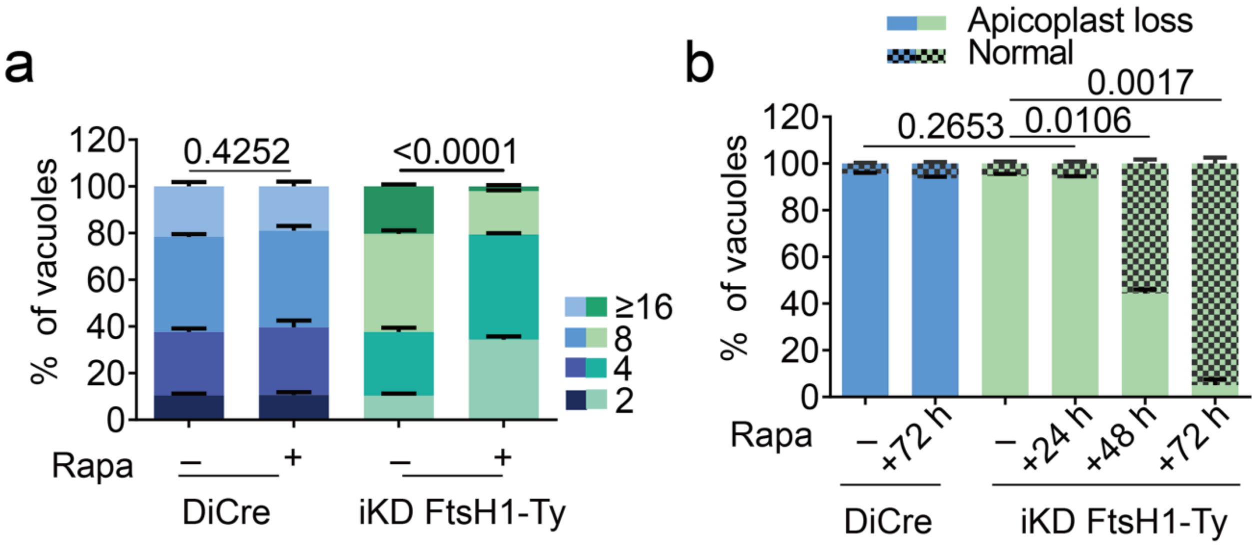

List of abbreviations used in main Figure 7c:

PBGS, porphobilinogen synthase; PBGD, porphobilinogen deaminase; UROS, uroporphyrinogen III synthase; UROD, uroporphyrinogen III decarboxylase; DXS, deoxy-d-xylulose 5-phosphate (DXP) synthase; DXR, DXP reductase; IspD, 2-C-Methyl-D-erythritol-4-phosphate cytidyltransferase; IspE, 4-diphosphocytidyl-2-C-methyl-D-erythritol kinase; IspF, 2-C-Methyl-d-erythritol 2,4-cyclodiphosphate synthase; IspG, 4-hydroxy-3-methylbut-2-en-1-yl diphosphate synthase; IspH, 4-hydroxy-3-methylbut-2-enyl diphosphate reductase; TPI2, triosephosphate isomerase 2; GAPDH, glyceraldehyde 3-phosphate dehydrogenase 2; PGK, phosphoglycerate kinase; APT, apicoplast phosphate translocator; PYK2, pyruvate kinase 2; PDHC, pyruvate dehydrogenase complex; ACC1, acetyl-coA carboxylase 1; ACP, acyl carrier protein; FabD, malonyl CoA-ACP transacylase; FabH, β-ketoacyl-ACP synthase III; FabG, 3-ketoacyl-ACP reductase; FabZ, 3-hydroxyacyl-ACP dehydratase; FabI, enoyl-ACP reductase; FabB/F, 3-oxoacyl-ACP synthase 1/2; LipB, lipoate protein ligase B; LipA, lipoyl synthase; FDR, ferredoxin—NAD(P)(+) reductase; FNR, ferredoxin-NADP(+) oxidoreductase; GdpA, glycerol 3-phosphate dehydrogenase; ATS1, glycerol 3-phosphate acyltransferase 1; ATS2, acyltransferase 2; NFS, cysteine desulfurase; SUF, iron sulfur cluster assembly protein; NFU, iron-sulfur scaffold homolog; GRXS, glutaredoxins; MEP/DOXP, 2-C-methyl-D-erythritol 4-phosphate/ 1-deoxy-D-xylulose 5-phosphate; FASII, type II fatty acid synthase. ALAS, aminolevulinic acid synthase; CPOX, coproporphyrinogen III oxidase; PPO, protoporphyrinogen oxidase; FECH, ferrochelatase; FPPS/GGPPS, bifunctional farnesyl diphosphate synthase/geranylgeranyl diphosphate synthase; COQ1, heptaprenyl diphosphate synthase; ELO, fatty acid elongase; KCR, 3-ketoacyl-CoA reductase; DEH, fatty acid dehydratase; ECR, 3-oxo-5-alpha-steroid 4-dehydrogenase; DES, fatty acyl-CoA desaturase.

Uncropped blots and images related to Supplementary Files

FigS1b integration PCR

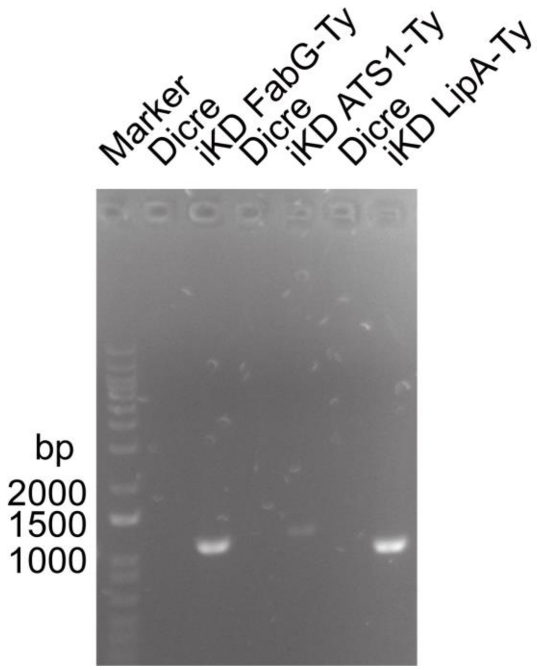

FigS3a integration PCR

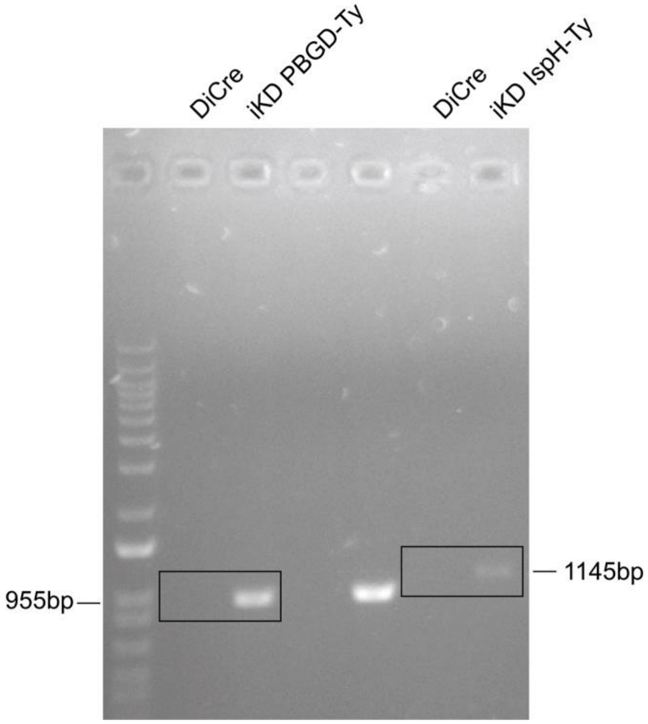

FigS3g integration PCR

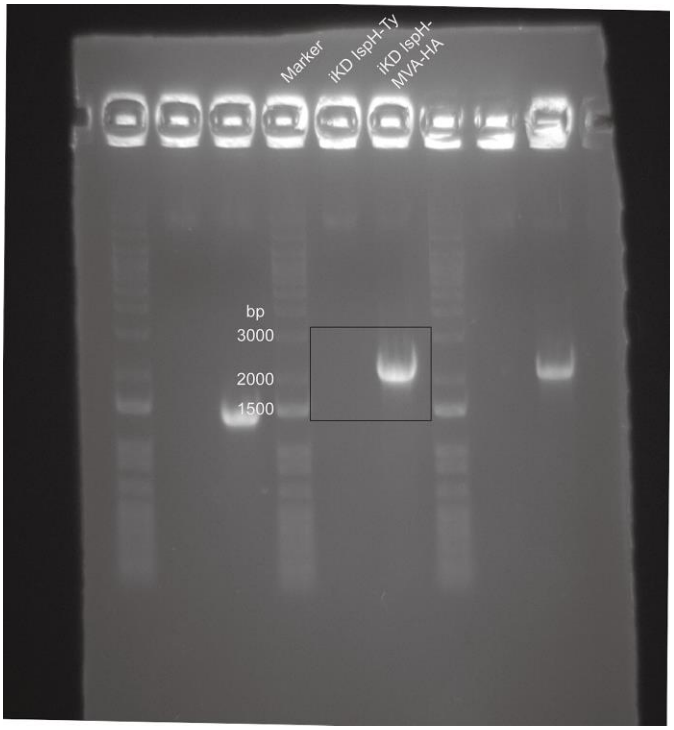

FigS4a integration PCR

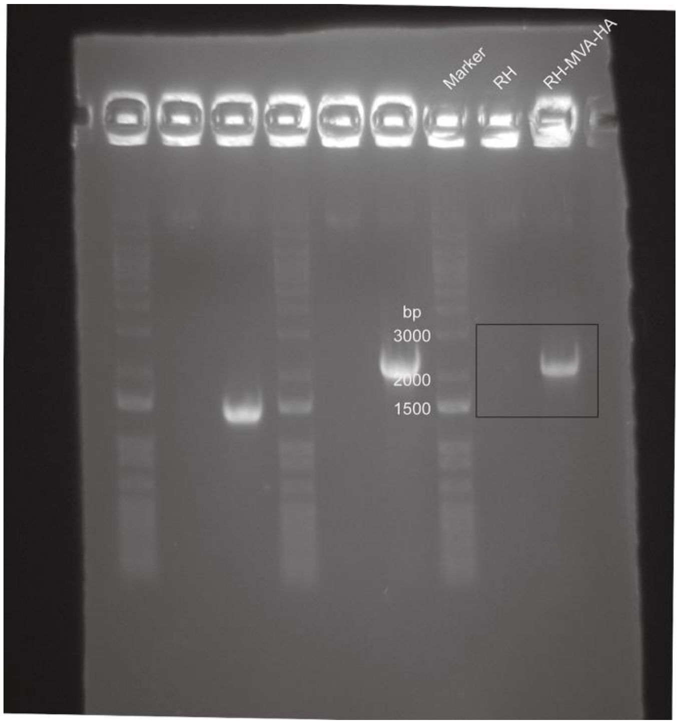

FigS4c western blot of MVA cassette

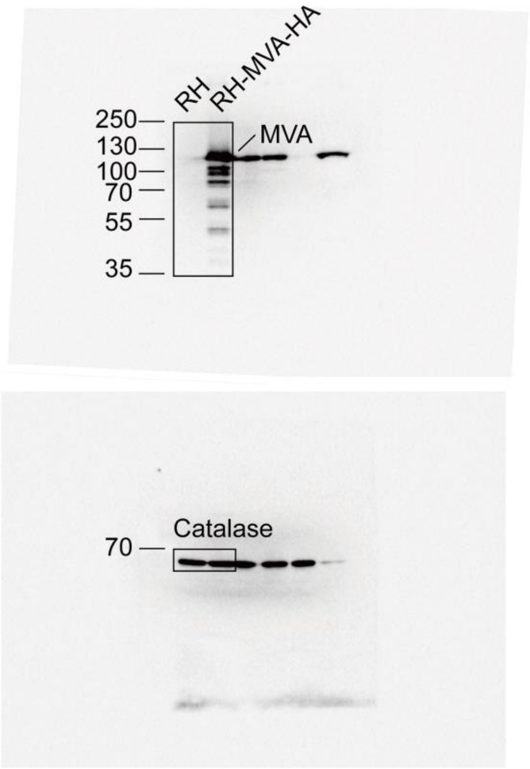

FigS4g western blot of mouse serum

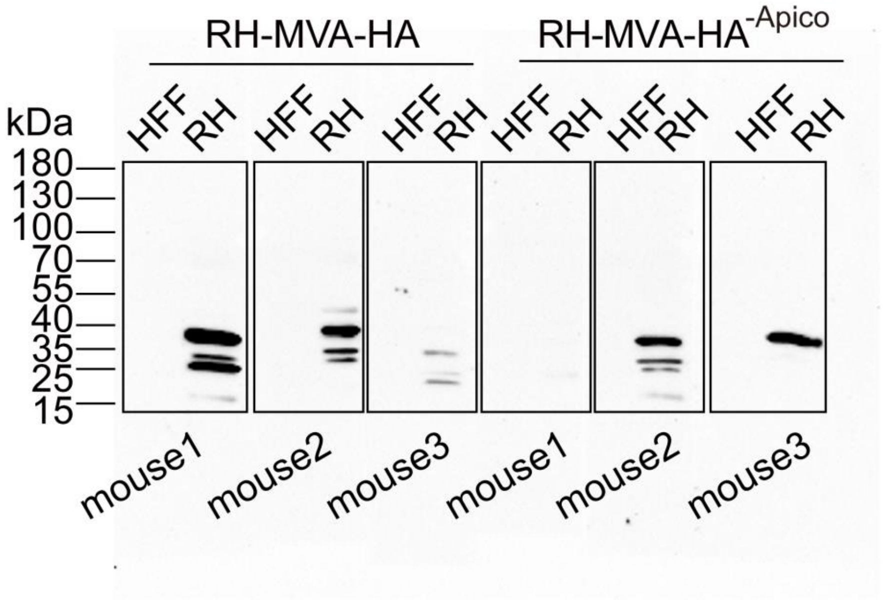

FigS6b UROS ko PCR

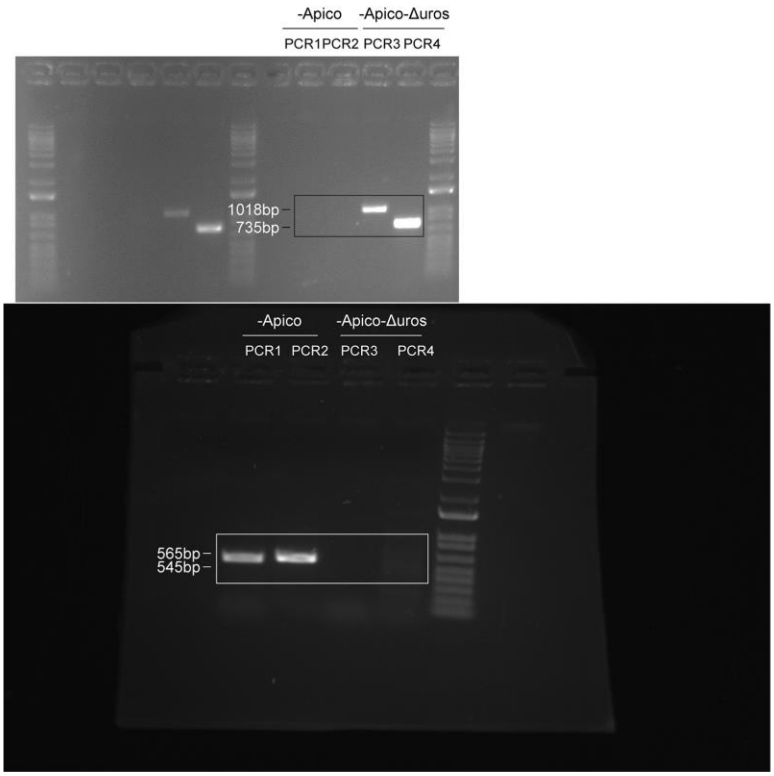

Supplement: Supplementary file 1 — Supplementary Information [file 41467_2025_57302_MOESM1_ESM.pdf]
