# Supplementary material for: Dissecting apicoplast functions through continuous cultivation of Toxoplasma gondii devoid of the organelle
Source: Nat Commun. 2025 Mar 1;16:2095. doi: 10.1038/s41467-025-57302-x (PMC11873192; doi:10.1038/s41467-025-57302-x)
Supplement: Supplementary file 2 — Description of Supplementary Files [file 41467_2025_57302_MOESM2_ESM.pdf]

Description of additional supplementary files

Title: Supplementary data 1: Description and sequences of all primers used in this study.

Description: Microsoft Word table listing the names and sequences of all oligonucleotides used in this study, including guide RNAs, integration PCR primers etc.

Title: Supplementary data 2: Analysis of fatty acid labeling in parasites depleted in LipA.

Description: Microsoft Excel file (one sheet) showing the area under the curve values for the M0 and M1 isotopologues of selected fatty acids (myristic acid, palmitic acid, stearic acid and oleic acid) measured by GC-MS, as well as the calculated percentage of ^{2}H-labelling and corrected isotopologue abundances. The corresponding results are plotted in Fig. 2g and the raw data has been deposited and can be accessed under the DOI: 10.26037/yareta:kc446nwxifbvtb5z7l2f6tayga. This data reveals a decrease in the fatty acid synthesis rate of parasites depleted in LipA.

Title: Supplementary data 3: Metabolomic profiling of parasites devoid of an apicoplast.

Description: Microsoft Excel file (four sheets) with metabolite abundances obtained by GC-MS analyses. The first sheet (All Metabolite Abundances) provides the retention time and quantifier ion for each metabolite as well as the corresponding abundances for all metabolites in all tested conditions (area under the curve). The second and third sheet provide the normalized signals, relative abundances and comparisons for the strains in normal medium and apicoplast rescue medium, respectively. The fourth sheet (Summary) shows the comparisons depicted in a heatmap equivalent to the data shown in Fig. 6a. The corresponding raw data has been deposited and can be accessed under the DOI: 10.26037/yareta:kc446nwxifbvtb5z7l2f6tayga. This data reveals differences in the abundance of metabolites depending on the presence or absence of the apicoplast.

Title: Supplementary data 4: Analysis of fatty acid labeling in parasites devoid of an apicoplast.

Description: Microsoft Excel file (two sheets) The first sheet (2H2O Intrc) of this file shows the area under the curve values for the M0 and M1 isotopologues of selected fatty acids (myristic acid and palmitic acid) measured by GC-MS, as well as the percentage of ^{2}H-labelling and corrected isotopologue abundances. The results are plotted in Fig. 6b. The corresponding raw data has been deposited and can be accessed under the DOI: 10.26037/yareta:kc446nwxifbvtb5z7l2f6tayga. This data determines the rate of fatty acid synthesis in intracellular parasites with or without an apicoplast cultured in normal medium or apicoplast rescue medium (24 hours labelling with 3.6% $^{2}H_2O$ v/v, while intracellular).

The second sheet (13C Extrc) of this Microsoft Excel file shows the area under the curve values for the M0-M14 isotopologues of myristic acid as measured by GC-MS, as well as the percentage of ^{13}C-labelling and corrected isotopologue abundances. The results are plotted in Fig. 6c. The corresponding raw data has been deposited and can be accessed under the DOI: 10.26037/yareta:lb356tpupbcfteq4t4ikalhapq. This data reveals the rate of fatty acid synthesis in

extracellular parasites with or without an apicoplast cultured in normal medium or apicoplast rescue medium (5 hours labelling with U-$^{13}C_6$-Glucose, while extracellular).

Title: Supplementary data 5: Proteomic analysis of parasites devoid of an apicoplast.

Description: This Microsoft Excel file (11 sheets) summarizes the data from the quantitative proteomic analysis of parasites that have an apicoplast or not and related comparisons. The file contains 11 sheets. Sheet 1 (All Comp) shows the comparison in the abundance of all proteins detected for all comparisons between the analyzed conditions. The corresponding Log2 ratios and Q (p-) values are given. Sheet 2 (Apico) compares the changes in protein abundance between parasites that have (RH-MVA, in apicoplast rescue medium, ARM) or not (RH-MVA-Apico, in apicoplast rescue medium, ARM) an apicoplast. All proteins that do not change (p-value >0.05 or fold change <1.5) are given as well as those that are significantly increased or decreased (p-value <0.05 and fold change >1.5). This provides the raw data underlying the volcano plot shown in Fig. 7a. Sheet 3 (Down No Apico) lists all proteins that are significantly downregulated in parasites without an apicoplast. The accession No, Log2 ratio, p-value, annotation, No. of transmembrane domains, presence of a signal peptide, fitness score and putative localization (LOPIT) are given for each of these proteins, as derived from ToxoDB. The tentative subcellular localization of the affected proteins is plotted in Fig. 3b. Sheet 4 (Down No Apico – Val LOPIT) lists the subset of proteins that are downregulated in parasites without an apicoplast and have been tentatively localized to the apicoplast. The same parameters as in sheet 3 are given for each protein. Sheet 5 (Down No Apico –LOPIT Other) lists the subset of proteins that are downregulated in parasites without an apicoplast and have been tentatively localized to cellular compartments other than the apicoplast. The same parameters as in sheets 3 and 4 are given for each protein. Note that red shading highlights affected proteins of the mitochondrial respiratory chain. Sheet 6 (Up No Apico) lists the subset of proteins that are upregulated in parasites without an apicoplast. The same parameters as in sheets 3, 4 and 5 are given for each protein. Sheet 7 (Metabolic Enzymes Behavior) summarizes the specific effect (up- or downregulated or unaffected) of known apicoplast metabolic proteins and related pathways in parasites without an apicoplast compared to those that have one. These results are graphically represented in Fig. 7c. Sheet 8 and 10 are equivalent to Sheet 2 but compare parasites that are cultured in ARM versus parasites that are cultured in normal medium (here: RegMed., RH-MVA in ARM vs normal medium, Medium, Sheet 8) and parasites that express the MVA cassette or not (RH vs RH-MVA, Strain, Sheet 10) Sheet 9 and 11 are equivalent to sheet 3 but listing proteins that are changing due to the culture media (RH-MVA in ARM vs normal medium, Medium Proteins, Sheet 9) or expression of the MVA cassette (RH vs RH-MVA, Strain Proteins, Sheet 11). The underlying raw data has been deposited in a data repository and can be accessed under the DOI: 10.26037/yareta:kc446nwxifbvtb5z7l2f6tayga. This dataset summarizes a comprehensive proteomic analysis, revealing changes in the proteome of parasites devoid of an apicoplast.

Title: Supplementary data 6: Transcriptomic analysis of parasites devoid of an apicoplast.

Description: This Microsoft Excel file (5 sheets) summarizes the data from the RNASeq analysis, comparing parasites that have an apicoplast (RH-MVA-HA in apicoplast rescue medium, ARM) or not (RH-MVA-HA-Apico in ARM). Sheet 1 (Raw Counts) shows the raw counts for each transcript. Sheet 2 (Normalized Counts) lists the normalized counts for each transcript. Sheet 3 (CompAll) shows the comparison between the two tested conditions, providing the fold-change and p-value for each transcript. Sheet 4 (SignificantlyChanged) lists all transcripts that change significantly (up- or downregulated; p-value <0.05 and fold change >1.5) in parasites that have an apicoplast

vs those that do not. The resulting volcano plot is given in Fig. 7d. For the affected genes, the accession No, Log2 ratio, p-value, annotation and putative localization (LOPIT) are given. Sheet 5 (ProteinRNASeqComp) compares genes that change significantly at the RNA and/or protein level, highlighting the overlap. Note that genes that change at both levels are highlighted with red shading. A volcano plot highlights the overlap between the two datasets is provided and shown in Fig. 7e. The corresponding raw data has been deposited and can be accessed under the DOI: 10.26037/yareta:lb356tpupbcfteq4t4ikalhapq. This dataset uncovers changes in the transcriptome upon loss of the apicoplast organelle.
