# Supplementary material for: Dissecting apicoplast functions through continuous cultivation of Toxoplasma gondii devoid of the organelle
Source: Nat Commun. 2025 Mar 1;16:2095. doi: 10.1038/s41467-025-57302-x (PMC11873192; doi:10.1038/s41467-025-57302-x)
Supplement: Supplementary file 3 — Supplementary Data 1. [file 41467_2025_57302_MOESM3_ESM.docx]

**Table S1.** Gene names and sequences of primers used in this study

| Construct | Number | Sequences | Description |  |
| --- | --- | --- | --- | --- |
|  | 1 | GATCTCGGTAGTCCAACTTGTgttttagagctagaaatagc | Guide RNA |  |
|  | 2 | GTGGACGGAGGCATGTTGTTTGGAGGCAACGTGGCGCatgcaTTGGAG | PCR homology1 |  |
| iKD FabG-Ty | 3 | ATCGCCTCCTCCACGTACATCCTCTCCAGACAGGAAACAGCTATGACCATG | PCR homology2 |  |
|  | 4 | GCTGTAGAGGTCGTCATGAGG | Forward primer to check integration |  |
|  | 5 | GCCGTAGTCTTCAATGGGTTTGG | Reverse primer to check integration |  |
| iKD ATS1-Ty | 6 | GCGTACAAAGCAGAAAGAGGTgttttagagctagaaatagc | Guide RNA |  |
|  | 7 | CTGTACAAACTGATCACTCCGCATTTCGAAGTGGCGCatgcaTTGGAG | PCR homology1 |  |
|  | 8 | ATATGTACACGTCAGCGAATCCCATCGACGCAGGAAACAGCTATGACCATG | PCR homology2 |  |
|  | 9 | GCGACACTCTAAGCGTCTTC | Forward primer to check integration |  |
|  | 10 | GTTCCAAGTGTGTAGAGGGAgttttagagctagaaatagc | Guide RNA |  |
|  | 11 | ACAACATTTCAAGTAAACGAGAAAGAGGCGGTGGCGCatgcaTTGGAG | PCR homology1 |  |
| iKD LipA-Ty | 12 | TGAAACTGGTTCCATGCGTCCAGCAGCGACCAGGAAACAGCTATGACCATG | PCR homology2 |  |
|  | 13 | TGATGATGCAAGGAAGCACG | Forward primer to check integration |  |
| iKD PBGD-Ty | 14 | GAAACGACGTTGCTCACCTGGgttttagagctagaaatagc | Guide RNA |  |
|  | 15 | ACTGAAGGTTGGAAGAATTTGAAAACGTTGGTGGCGCatgcaTTGGAG | PCR homology1 |  |
|  | 16 | CGACGTAGTTGACGATCGGTCGTGTCTGTACAGGAAACAGCTATGACCATG | PCR homology2 |  |
|  | 17 | AAGAAATCAAGAAGGCCGCG | Forward primer to check integration |  |
| iKD IspH-Ty | 18 | GCGTGTCTATGTACAAGACAGgttttagagctagaaatagc | Guide RNA |  |
|  | 19 | GGAGTGGAGACGCTGGTCGCCCGAGACTCTGTGGCGCatgcaTTGGAG | PCR homology1 |  |
|  | 20 | AACGTGTTTCTGCACCGCTGAGCAACAAGCCAGGAAACAGCTATGACCATG | PCR homology2 |  |
|  | 21 | GCTGATATGCAACTGCGTTCG | Forward primer to check integration |  |
| iIspH-MVA-HA/ RH-MVA-HA | 22 | CAGGGCTTCTAAAATGGCGCgttttagagctagaaatagc | Guide RNA1 |  |
|  | 23 | GCTCCCACGTCCCTCACCATgttttagagctagaaatagc | Guide RNA2 |  |
|  | 24 | CAAGGCGTATTCCTTTTTTCGTCGGACCTGTATAGGGCGAATTGGGTACC | PCR homology1 |  |
|  | 25 | CGCACCAGGCAGCCTCACAACTAAAACTTTGGCCGCTCTAGAACTAGTG | PCR homology2 |  |
|  | 26 | agcacctagactcgtcctgc | Forward primer to check MVA integration |  |
|  | 27 | AGGGTTCGCGTTGGGGTT | Reverse primer to check MVA integration |  |
| Apicoplast genome | 28 | CACAAATGGATGGAGCAATCTT | Forward primer to check apicoplast genome |  |
|  | 29 | TCAATGGTAGAGCAAAGGACTG | Reverse primer to check apicoplast genome |  |
| Actin | 30 | ATGGCGGATGAAGAAGTGCAAG | Forward primer to check Actin |  |
|  | 31 | ATGTCGTCCCAGTTGGTGAC | Reverse primer to check Actin |  |
|  | 32 | AACAACGTGTTCGCTCCTCG | Guide RNA1 |  |
|  | 33 | GTGCACAAAGGTCTCTCCTT | Guide RNA2 |  |
|  | 34 | TTTCACCCTTTTCCCTCTCACCTGTGTTCTgcggccgctctagaactag | PCR Homology 1 |  |
|  | 35 | CCTCAAATCGGAGACGGGACCGAGAAGAGAgcggaagatccgatcttgc | PCR Homology 2 |  |
|  | 36 | TGCTGGACATTCCCTGCTCG | Fw integration primer in 5’ UTR |  |
| *uros*-KO (DHFR) | 37 | GCGGACAGAGAAGATACCAGC | Rv integration primer in 5’ coding region |  |
|  | 38 | GCCACAATATCGTCATGAAGGTGC | Fw integration primer in 3’ coding region |  |
|  | 39 | GGATAAGCGCGAGAAAGAGGC | Rv integration primer in 3’ UTR |  |
|  | 40 | GTCACTTGTTGTGCCAGTTCTAC | Rv integration primer in 5’ DHFR |  |
|  | 41 | CTTGGGGGTCATCGCGACGACCAGAC | Fw integration primer in 3’ DHFR |  |
|  | 42 | GCGAAGCAGAAAACTTAACCAgttttagagctagaaatagc | Guide RNA |  |
| iKD FtsH1-Ty | 43 | TTGGGGCTATCCACTATTACGCGAGACGAAGTGGCGCatgcaTTGGAG | PCR homology1 |  |
|  | 44 | ATAGGAGGCTTCCCATGGACATGATAACCTCAGGAAACAGCTATGACCATG | PCR homology2 |  |
|  | 45 | GTCACACACGTCACTTAAGCAgttttagagctagaaatagc | Guide RNA |  |
| iKD 201270-Ty | 46 | CAGAGACTGAAAGATAATCAGTTGTTTGGTGTGGCGCatgcaTTGGAG | PCR homology1 |  |
|  | 47 | TGCACCCTTGAGTATTGCTACAAACTAACACAGGAAACAGCTATGACCATG | PCR homology2 |  |
| iKD 248770-Ty | 48 | AAGTTacccggtgcgcagctgcgaaG | Forward primer for guide RNA |  |
|  | 49 | AAAACttcgcagctgcgcaccgggtA | Reverse primer for guide RNA |  |
|  | 50 | GACGACGACCTCAGTGGTCCACTCGACGACGTGGCGCatgcaTTGGAG | PCR homology1 |  |
|  | 51 | gctcgttatatacaactacttgtccctcgcCAGGAAACAGCTATGACCATG | PCR homology2 |  |
