# Supplementary material for: Dissecting apicoplast functions through continuous cultivation of Toxoplasma gondii devoid of the organelle
Source: Nat Commun. 2025 Mar 1;16:2095. doi: 10.1038/s41467-025-57302-x (PMC11873192; doi:10.1038/s41467-025-57302-x)
Supplement: Supplementary file 9 — Reporting summary [file 41467_2025_57302_MOESM9_ESM.pdf]

nature portfolio

Corresponding author(s): Hong-Juan Peng
Dominique Soldati-Favre
Joachim Kloehn

Last updated by author(s): 2025.02.13

Reporting Summary

Nature Portfolio wishes to improve the reproducibility of the work that we publish. This form provides structure for consistency and transparency in reporting. For further information on Nature Portfolio policies, see our Editorial Policies and the Editorial Policy Checklist.

Statistics

For all statistical analyses, confirm that the following items are present in the figure legend, table legend, main text, or Methods section.

n/a	Confirmed	
☐	☒	The exact sample size (*n*) for each experimental group/condition, given as a discrete number and unit of measurement
☐	☒	A statement on whether measurements were taken from distinct samples or whether the same sample was measured repeatedly
☐	☒	The statistical test(s) used AND whether they are one- or two-sided<br>*Only common tests should be described solely by name; describe more complex techniques in the Methods section.*
☒	☐	A description of all covariates tested
☐	☒	A description of any assumptions or corrections, such as tests of normality and adjustment for multiple comparisons
☐	☒	A full description of the statistical parameters including central tendency (e.g. means) or other basic estimates (e.g. regression coefficient) AND variation (e.g. standard deviation) or associated estimates of uncertainty (e.g. confidence intervals)
☐	☒	For null hypothesis testing, the test statistic (e.g. *F*, *t*, *r*) with confidence intervals, effect sizes, degrees of freedom and *P* value noted<br>*Give P values as exact values whenever suitable.*
☒	☐	For Bayesian analysis, information on the choice of priors and Markov chain Monte Carlo settings
☒	☐	For hierarchical and complex designs, identification of the appropriate level for tests and full reporting of outcomes
☒	☐	Estimates of effect sizes (e.g. Cohen's *d*, Pearson's *r*), indicating how they were calculated

Our web collection on statistics for biologists contains articles on many of the points above.

Software and code

Policy information about availability of computer code

Data collection	Data acquisition was performed using Biorad ImageLab, ImageJ (FIJI), Olympus iTEM, Zeiss ZEN, Agilent MassHunter (Quantitative Analysis and Qualitative Analysis 10.0), Thermo Scientific XCalibur
Data analysis	Data analysis was performed using ImageJ (FIJI), Microsoft Excel, Graphpad Prism 10, Agilent MassHunter (Quantitative Analysis and Qualitative Analysis 10.0), Spectronaut v.18 (Biognosys)

For manuscripts utilizing custom algorithms or software that are central to the research but not yet described in published literature, software must be made available to editors and reviewers. We strongly encourage code deposition in a community repository (e.g. GitHub). See the Nature Portfolio guidelines for submitting code & software for further information.

Data

Policy information about availability of data

All manuscripts must include a data availability statement. This statement should provide the following information, where applicable:

- Accession codes, unique identifiers, or web links for publicly available datasets
- A description of any restrictions on data availability
- For clinical datasets or third party data, please ensure that the statement adheres to our policy

All data and accession codes are presented in the manuscript. No restrictions apply.

Source data is included in the Source Data file. All supplementary data is included in the Supplementary File and the Supplementary Data 1-6. Raw data files can be accessed from a public repository (Yareta) that operates under the FAIR principles using the following DOI: 10.26037/yareta:kc446nwxifbvtb5z7l2f6tayga or the URL: https://doi.org/10.26037/yareta:kc446nwxifbvtb5z7l2f6tayga
And under the DOI: 10.26037/yareta:lb356tpupbcfteq4t4ikalhapq or the URL: https://doi.org/10.26037/yareta:lb356tpupbcfteq4t4ikalhapq
All raw data is available and freely accessible for a minimum of 10 years.

Research involving human participants, their data, or biological material

Policy information about studies with human participants or human data. See also policy information about sex, gender (identity/presentation), and sexual orientation and race, ethnicity and racism.

Reporting on sex and gender	N/A
Reporting on race, ethnicity, or other socially relevant groupings	N/A
Population characteristics	N/A
Recruitment	N/A
Ethics oversight	N/A

Note that full information on the approval of the study protocol must also be provided in the manuscript.

Field-specific reporting

Please select the one below that is the best fit for your research. If you are not sure, read the appropriate sections before making your selection.

☒ Life sciences ☐ Behavioural & social sciences ☐ Ecological, evolutionary & environmental sciences

For a reference copy of the document with all sections, see nature.com/documents/nr-reporting-summary-flat.pdf

Life sciences study design

All studies must disclose on these points even when the disclosure is negative.

Sample size	Experiments were designed to include all necessary controls and sufficient sample size for statistical analyses (minimum of independent biological triplicates). Sample size for mice experiment was selected to provide statistically meaningful data but reduce number of animals used (3R principle). The selected samples sizes are according to the standards in the field as used in numerous other studies, providing sufficient statistical power:<br><br>Krishnan, A. et al. Functional and Computational Genomics Reveal Unprecedented Flexibility in Stage-Specific Toxoplasma Metabolism. Cell Host Microbe 27, 290-306 e211 (2020). https://doi.org/10.1016/j.chom.2020.01.002<br><br>Alberione, M. P. et al. N-acetylglucosamine supplementation fails to bypass the critical acetylation of glucosamine-6-phosphate required for Toxoplasma gondii replication and invasion. PLoS Pathog 20, e1011979 (2024). https://doi.org/10.1371/journal.ppat.1011979
Data exclusions	No data was excluded from the presented findings
Replication	All experiments were conducted with a minimum of three independent biological replicates - the values for each replicate are presented. All presented findings were reproducible.
Randomization	All mass Spectrometry Samples for Metabolomic and Proteomic Analyses were analyzed in randomized sequences
Blinding	Blinding was used for the unbiased quantification of IFAs (Growth Assays and Apicoplast Loss Assays) and plaque assay experiments

Reporting for specific materials, systems and methods

We require information from authors about some types of materials, experimental systems and methods used in many studies. Here, indicate whether each material, system or method listed is relevant to your study. If you are not sure if a list item applies to your research, read the appropriate section before selecting a response.

Materials & experimental systems

n/a	Involved in the study
☐	☒ Antibodies
☐	☒ Eukaryotic cell lines
☒	☐ Palaeontology and archaeology
☐	☒ Animals and other organisms
☒	☐ Clinical data
☒	☐ Dual use research of concern
☒	☐ Plants

Methods

n/a	Involved in the study
☒	☐ ChIP-seq
☒	☐ Flow cytometry
☒	☐ MRI-based neuroimaging

Antibodies

Antibodies used	All antibodies are listed in the methods section of the manuscript and here: The primary antibodies for IFAs were as follows: anti-GAP45 (1:10,000), anti-actin (1:20), anti-Ty (1:10, BB2), anti-Cpn60 (1:3,000), anti-ATrx1 (1:3,000), anti-HA (1:10, BB2). The secondary antibodies for IFAs were as follows: anti-mouse 488 (1:3,000, Invitrogen A11001), anti-rabbit Alexa fluor 594 (1:3,000, Invitrogen A11012). The primary antibodies for western blot were as follows: anti-catalase (1:2,000), anti-Ty (1:10, BB2), anti-Cpn60 (1:3,000), anti-HA (1:1,000, Sigma-Aldrich,H6908), anti-IMC1 (1:1,000). The secondary antibodies for western blot were as follows: anti-mouse HRP (1:3,000, Sigma-Aldrich, A5278), anti-rabbit HRP (1:3,000, Sigma-Aldrich, A8275).<br>All details regarding the source and validation of these antibodies can be obtained from the studies cited in the Methods section (Reagents and antibodies) of this manuscript.
Validation	Antibodies were of commercial origin or validated in previous studies (see citations in the manuscript - Methods section: Reagents and antibodies).

Eukaryotic cell lines

Policy information about cell lines and Sex and Gender in Research

Cell line source(s)	Human Foreskin Fibroblasts: ATCC SCRC-1041
Authentication	No authentication for the mammalian cell line
Mycoplasma contamination	All cell lines tested negative for Mycoplasma
Commonly misidentified lines<br>(See ICLAC register)	N/A

Animals and other research organisms

Policy information about studies involving animals; ARRIVE guidelines recommended for reporting animal research, and Sex and Gender in Research

Laboratory animals	This study used 7 weeks old female B6CBAF1/J mice. Note that this was reported differently in a previous version of the manuscript and was corrected here.
Wild animals	N/A
Reporting on sex	Experiments were performed with female mice
Field-collected samples	N/A
Ethics oversight	All animal experiments were conducted with the authorization numbers GE125A, according to the guidelines and regulations issued by the Swiss Federal Veterinary Office.

Note that full information on the approval of the study protocol must also be provided in the manuscript.

Plants

Seed stocks	N/A
Novel plant genotypes	N/A
Authentication	N/A
